# Synthesis and Antimicrobial Activity of Some New Quinoxaline Derivatives

**DOI:** 10.3390/ph3082416

**Published:** 2010-07-30

**Authors:** Dharmchand Prasad Singh, Sanjay Kumar Deivedi, Syed Riaz Hashim, Ram Gopal Singhal

**Affiliations:** 1 Department of Pharmaceutical Chemistry, College of Pharmacy, Institute of Foreign Trade and Management, Moradabad - 244001, India; E-Mails: sanjaydeivedi@yahoo.co.in (S.K.D.); sriaz_hashim@yahoo.com (S.R.H.); 2 School of Pharmaceutical Sciences, Shobhit University, Meerut, India; E-Mail: drrgsinghal@rediffmail.com (R.G.S.)

**Keywords:** quinoxalines, benzamine, benzaldehyde, antimicrobial activity

## Abstract

2-Chloro-3-methylquinoxaline was selected as a nucleus around which various molecular transformations were performed to obtain new compounds expected to possess optimized antimicrobial activity. As very little work regarding attachment of ether linkages replacing chlorine at C-2 has been reported, it was thought worthwhile to synthesize various quinoxaline derivatives by replacing the C_2_ chlorine with an ether linkage attached to a benzene ring possessing an aldehyde or a free amino group which can be further reacted with aromatic amines and aromatic aldehydes, respectively, to yield new Schiff bases containing quinoxaline moieties. Thus the compounds 4-(2-methylquinoxalinyloxy) benzaldehyde (**4**), 2-[4-(substituted-benziminomethyl)-phenoxy]-3-methyl quinoxalines **5a–e**, 4-(2-methyl-quinoxaline-3-yloxy)benzamine (**6**) and 4-(2-methylquinoxalin-3-yloxy)-*N*-substituted benzylidine benzamines **7a–e** were synthesized and tested for their antimicrobial activity. The structures of the compounds were confirmed on the basis of their elemental and spectral data.

## 1. Introduction

Compounds containing the quinoxaline nucleus exhibit a broad spectrum of biological activity such as antibacterial [1,2,3], antifungal [4,5], antiviral [6], anticancer [7], antituberculosis [8], antimalarial [9] and anti-inflammatory properties [10]. Many researchers have reported the synthesis and biological activity of quinoxaline derivatives [11,12,13,14]. In the light of these facts we decided to synthesize some new quinoxaline derivatives incorporating aromatic aldehyde and aromatic amine moieties attached to a 2-hydroxy-3-methylquinoxaline nucleus with an ether linkage followed by the treatment with aromatic amines or aromatic aldehydes to afford Schiff bases in the hope of obtaining better antimicrobial agents. All the synthesized compounds were screened for their antimicrobial activity.

## 2. Result and Discussion

### 2.1. Synthesis

**Scheme 1:**
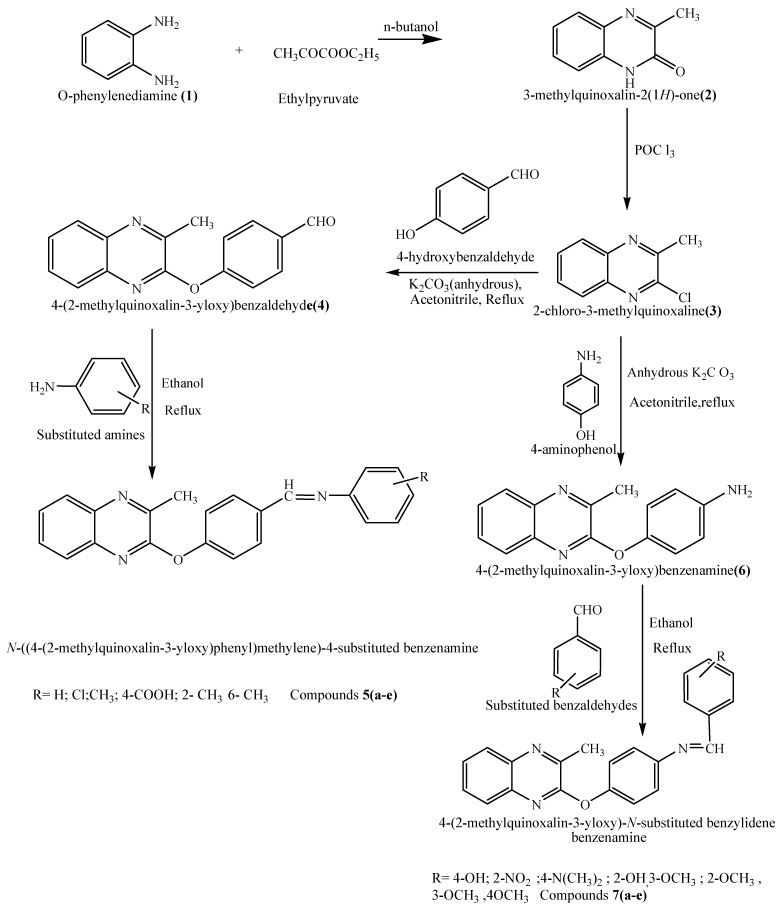
Synthesis of some new quinoxaline derivatives.

The chemical synthesis (Scheme 1) was initiated with the reaction of *o*-phenylenediamine (**1**) with ethyl pyruvate in *n*-butanol to yield 2-hydroxy-3-methylquinoxaline (**2**), which on treatment with POCl_3_ yielded 2-chloro-3-methylquinoxaline (**3**). A mixture of compound **3** and 4-hydroxy benzaldehyde was next refluxed in acetonitrile for 30 hours to afford 2-(*p*-formylphenoxy)-3-methyl quinoxaline (**4**) as an intermediate. Mixtures of compound **4** and various substituted aromatic amines were refluxed in ethanol to afford 2-[4-(substituted benziminomethyl)phenoxy]-3-methylquinoxalines **5a–e**. In another set of reactions, compound **3** was refluxed with *p*-aminophenol in acetonitrile for 30 hours to yield 4-(2-methylquinoxalin-3-yloxy) benzamine (**6**) as a second intermediate. Compound **6** was refluxed with different substituted aromatic aldehydes in order to prepare compounds **7a–e**. The structures of all newly synthesized compounds were elucidated on the basis of their spectral and analytical data.

The IR spectrum of compound **4** showed absorption bands at 3,038 cm^−1 ^due to CH_3_ stretching, at 1,600 cm^−1^ due to C=N stretching, a strong band at 1,699 cm^−1 ^due to an aldehyde function and a band at 1,222 cm^−1 ^due to the C-O-C aryl ether (C-O stretching). Its ^1^H-NMR spectrum showed a singlet (3H) at δ 2.870 due to CH_3_ protons, a broad set of multiplets between δ 6.6 - 8.0 (8H) due to aromatic hydrogens and a sharp singlet at δ 10.07 due to an aldehyde proton. This indicated that a free aldehyde function was present which could be reacted with substituted aromatic amines to form Schiff bases. Similarly the IR spectrum of compound **6** showed two bands at 3,466 cm^−1 ^and 3,425 cm^−1 ^due to primary amine N-H stretching, while other bands at 3,041 cm^−1^ due to CH_3_ stretching and a band at 1,220 cm^−1^ due to (C-O-C) aryl ether were also present. In the case of the intermediate **6**, the ^1^H-NMR spectrum showed a sharp singlet at δ 2.825 due to the protons of a CH_3_ group attached to the quinoxaline ring, a broad D_2_O exchangeable singlet at δ 3.742 due to NH_2_ protons, and a characteristic aromatic proton multiplet between δ 6.77-8.00 ppm. A singlet at around δ 8.40-9.03 due to presence of the (**CH**=N-) group in the compounds **5a–e** and **7a–e** clearly suggested the formation of the expected Schiff bases. IR, ^1^H-NMR spectra and elemental analytical data of compounds **5a–e** and **7a–e** confirmed the structures of the newly synthesized compounds.

### 2.2. Antibacterial Activity

The antibacterial activity was determined by the disc diffusion method at the concentration of 50 µg per disk. All the synthesized compounds were tested *in vitro* for their antibacterial activity against microorganisms such as *Staphylococcus aureus*, *Bacillus subtilis* (Gram positive), *Escherichia coli*, *Pseudomonas aeruginosa* (Gram negative), using ciprofloxacin as standard antibacterial. The results of activity, presented in the Table 1 suggested that compounds **4**, **5a**, **5c**, **5d**, **5e**, **7a** and **7c** were highly active against both Gram positive and Gram negative bacteria, among them compounds **5c**, **5d**, **7a**, **7c**, and **7e** were specifically highly active against *E. coli*. Compound **5a** possessed no activity against *Bacillus subtilis*, Compounds **5b**, and **5c** showed no activity against *Pseudomonas aeruginosa.* Compound **7d** showed no activity against *Escherichia coli and Pseudomonas aeruginosa.* The rest of tested compounds were found to have moderate antibacterial activity. The high activity of compounds **4**, **5a**, **5c**, **5d**, **5e** and **7c** could be explained on the basis of the contributions of incorporated aromatic ring and –CH_3_ groups, which we know should increase the lipophilicity of the compounds. This increase in lipophilicity would help their permeability through the microbial cell wall resulting in higher activity. Compound **5d** may be considered the analogue of benzoic acid (a known antimicrobial) due the presence of the –COOH group. 

### 2.3. Antifungal Activity

The antifungal activity was tested against strain such as *A. niger* and *C. albicans*, using fluconazole as standard antifungal. Compounds **4**, **6**, and **7a** showed moderate activity against both strains. Compounds **5a**, **5b**, **5c**, **5d** and **5e** showed no activity against *A. niger*. Compounds **7b**, **7c**, **7d** and **7e** showed moderate activity against *A. niger* but no activity against *C. albicans.*

**Table 1:** Results of antimicrobial activity of the compounds, zones of inhibition (in mm).

Compounds	Zone of Inhibition					
	*S. aureus* (NCIM 2079)	*B. subtilis* (NCIM 2439)	*E. coli* (NCIM 2831)	*P. aerug.* (NCIM 2863)	*A. niger* (NCIM 618)	*C. alb.* *(NCIM 3557)*
**4**	+ + +	+ +	+ +	+ +	+ +	+ +
**5a**	+ +	-	+ + +	+ +	-	-
**5b**	+ +	+ +	+ +	-	-	-
**5c**	+ + +	+ +	+ + +	-	-	+ +
**5d**	+ + +	+ + +	+ + +	+ +	-	-
**5e**	+ + +	+ +	+ +	+ +	-	+ +
**6**	+ +	+ +	+ +	+ +	+ +	+ +
**7a**	+ + +	+ + +	+++	++	+ +	+ +
**7b**	+ +	+ +	+ +	+ +	+ +	-
**7c**	+ + +	+ +	+ + +	+ +	+ +	-
**7d**	+ +	+ +	-	-	+ +	-
**7e**	+ +	+ +	+ + +	+ +	+ +	-
**Ciprofloxacin**	+ + +	+ + +	+ + +	+ + +		
**Fluconazole**					+ + +	+ + +

Key to symbols: - inactive (inhibition zone < 6 mm); slightly active = + (inhibition zone 7–9 mm); moderately active = + + (inhibition zone 10-13 mm); highly active = + + + (inhibition zone > 14 mm).

## 3. Experimental

### 3.1. General

All recorded melting points were determined on a laboratory melting point apparatus using the capillary method and are uncorrected. Purity of the compounds was checked by thin layer chromatography using silica gel-G on micro slide glass plates and spots were detected under iodine vapor. IR spectra were recorded in KBr disk on a Shimadzu FTIR-8400 spectrophotometer and ^1^H-NMR spectra on a JEOL FT-NMR Spectrometer (300 MHz) using TMS as an internal standard. All chemical shift values were recorded as δ (ppm).

#### 3.1.1. Synthesis of 3-methylquinoxalin-2-(1H)-one (**2**)

o-Phenylenediamine (10.8 g, 0.10 M) was dissolved in *n*-butanol (300 mL) with warming. Ethyl pyruvate (11.6 g, 15 mL, 0.10 M) was dissolved separately in *n*-butanol (100 mL) and added to the former solution with constant stirring. The solution was set aside for 30 min, and then it was heated for 1 hour on a water bath. On cooling, the crystals that separated were filtered, washed with *n*-hexane and purified by recrystallization from ethanol to yield colorless, needle-shaped crystals of 2-hydroxy-3-methylquinoxaline. Yield 80%; m.p. 246 °C ([15,16] m.p. 245 °C); IR (KBr, cm^−1^): 3,008 (2° amide N–H), 2,968 (methyl group C-H), 1,665 (amide C=O), and 1,610 (aromatic nucleus C=C multiple bond).

#### 3.1.2. Synthesis of 2-chloro-3-methylquinoxaline (**3**)

2-Hydroxy-3-methylquinoxaline (16.0 g, 0.10 M) in POCl_3_ (60 mL) was refluxed for 90 min. Then the excess of POCl_3_ was distilled off and the residue was cooled to room temperature and added to crushed ice taken in a 1 L beaker. The mixture was made alkaline by adding 2% NaOH solution to isolate the product. The crude product was recrystallized from petroleum ether (40–60 °C), to yield the crystals of 2-chloro-3-methylquinoxaline (**3**). Yield 60%; m.p. 88 °C (lit. [17] m.p. 86–87 °C); IR (KBr) data clearly showed the disappearance of the amide C=O stretching vibration and the appearance of an aryl halide C-Cl stretching vibration at 1,038.52 cm^−1^.

#### 3.1.3. Synthesis of 4-(2-methylquinoxalinyloxy) benzaldehyde (**4**)

*p*-Hydroxybenzaldehyde (0.1.22 g, 0.01 M) was dissolved in acetonitrile (50 mL) taken in a 250 mL round bottomed flask. Anhydrous K_2_CO_3_ (2.0 g) was added to the mixture, which was refluxed for 1 hour, then 2-chloro-3-methylquinoxaline (1.785 g, 0.01 M) was added and the mixture was further refluxed for 30 hours. At the end of the reaction time, the mixture was filtered and excess of acetonitrile was distilled off to get the product. The crude product was treated with 2%NaOH solution to dissolve any unreacted *p*-hydroxybenzaldehyde. The product was filtered, washed with distilled water to remove traces of alkali and recrystallized from ethanol to get crystals of 4-(2-methylquinoxalinyloxy) benzaldehyde (**4**). Yield 70%; m.p. (116–117 °C); IR (KBr, cm^−1^): 1,736 (aldehyde C=O), 1,222 (ether C-O); ^1^H-NMR (CDCl_3_): 2.880 (s, 3H, CH_3_ attached to a quinoxaline ring), 7.288–8.047 (m, 8H, aromatic protons); 10.076 (s, 1H, aldehyde proton). 

### 3.2. General procedure for the synthesis of 2-[4-(substituted benziminomethyl) phenoxy]-3-methyl-quinoxalines *5a–e*

Compound **4** (0.01 M) and substituted aromatic amines (0.01 M) in ethanol (25mL) containing a catalytic amount of glacial acetic acid were refluxed for an appropriate time to complete the reaction, as monitored by TLC. After completion of the reactions, the flask contents were cooled and the separated crystalline compound was filtered, washed with a little ethanol and recrystallized from ethanol.

#### 3.2.1. *N*-[(4-(2-Methylquinoxalin-3-yloxy)-phenyl)-methylenebenzamine (**5a**)

IR (KBr, cm^−1^): 3,060 (methyl C-H), 1,578 (-C=N-), 1,222 (ether C-O); ^1^H-NMR (CDCl_3_): 2.843 (s, 3H, CH_3_ attached to quinoxaline ring), 7.226–8.033 (m, 13H, Ar-H), 8.507 (s, 1H, CH=N-); MS: m/z 339. Anal. calc. for C_22_ H_17_ N_3_ O: C 77.86, H 5.05, N 12.38%. Found: C 77.65, H 5.20, N 12.52%.

#### 3.2.2. [(4-(2-Methylquinoxalin-3-yloxy)-phenyl)-methylene)-2-chlorobenzamine (**5b**)

IR (KBr, cm^−1^) 3,059, (methyl C-H), 1,583, (-C=N-), 1,222, (ether C-O) and 1,038, (aryl C-Cl). ^1^H NMR (CDCl_3_): 2.847, (s, 3H, CH_3_ attached to quinoxaline ring), 7.044–8.073(12H, Ar-H), 8.437, (s, 1H, CH=N-). MS: m/z 373. Anal. Calculated for C_22_ H_16_ Cl N_3_ O: C 70.68, H 4.31, N 11.24%.Found: C 70.75, H 4.20, N 11.46%.

#### 3.2.3. *N*-[{4(2-Methylquinoxalin-3-yloxy)-phenyl}-methylyne]-4-methylbenzeneamine (**5c**)

IR (KBr, cm^−1^): 1,578 (-C=N), 1,223 (ether C-O). Disappearance of band at 1,735.85 for aldehyde indicated the formation of –CH=N- group.^1^H NMR (CDCl_3_): 2.838 (s, 3H, CH_3_ attached to quinoxaline ring), 2.387 (s, 3H, CH_3_ attached to benzene), 7.148–8.018 (m, 12H, Ar-H) 8.511, (s, 1H, CH=N-).MS: m/z 353. Anal. Calculated for C_23_ H_19_ N_3_ O: C 78.16, H 5.42, N 11.89%.Found: C 78.08, H 5.54, N 11.78%.

#### 3.2.4. 4-((4-(2-Methylquinoxalin-3yloxy)-phenyl) - methylene-aminobenzoicacid (**5d**)

IR (KBr, cm^−1^) 2,927 (methyl C-H), 1,686 (carboxylic C=O), 1,583 (-C=N-), 1,222 (ether C-O). ^1^H NMR (DMSO): 2.767 (s, 3H, CH_3_ attached to quinoxaline ring), 7.121–8.094(m, 12H, Ar-H), 8.686, (s, 1H, CH=N-), 12.836 (s, 1H, COOH). MS: m/z 383. Anal. Calculated for C_23_ H_17_ N_3_ O_3_: C 72.05, H 4.47, N 10.96%.Found: C 71.95, H 4.22, N 11.00%.

#### 3.2.5. *N*-[(2-methylquinoxalin-3-yloxy)-phenyl)-methylene)-2, 6-dimethylbenzamine (**5e**)

IR (KBr, cm^−1^) 2,919 (methyl C-H), 1,577 (-C=N-), 1,224 (ether C-O). ^1^H NMR (CDCl_3_): 2.846(s, 3H, CH_3_ attached to quinoxaline ring), 2.336 and 2.317 (d, 6H, CH_3_ on aromatic benzene), 6.803–8.042 (m, 11H, Ar-H) 8.390, (s, 1H, CH=N-) group. MS: m/z 367. Anal. Calculated for C_24_ H_21_ N_3_ O: C 78.45, H 5.76, N 11.44%.Found: C 78.62, H 5.58, N 11.52%.

### 3.3. Synthesis of 4-(2-methylquinoxalin-3-yloxy)-benzamine *(**6**)*

4-Aminophenol (1.09 g, 0.01 M) was dissolved in a mixture of acetonitrile (50 mL) and DMF (10 mL) containing anhydrous K_2_CO_3_ (2.0 g). The mixture was refluxed for 1 hour then of 2-chloro-3-methylquinoxaline (1.785 g, 0.01 M) was added and mixture was further refluxed for 30 hour. The mixture was filtered and the excess of acetonitrile was distilled off to get the product. The crude product was treated with 2% NaOH solution to dissolve any unreacted 4-aminophenol. The product was filtered, washed with distilled water to remove traces of alkali and recrystallized from ethanol to give crystals of 4-(2-methylquinoxaline-3yloxy)-benzamine, yield 65%; m.p. 178 °C; IR (KBr, cm^−1^): 3,466 and 3,426 (1° amine group N-H), 3,041 (methyl group C-H), 1,620 (C=N) and 1,221 (ether C-O); ^1^H-NMR (CDCl_3_): 2.825 (s, 3H, CH_3_ attached to quinoxaline ring), 3.742(s, 2H, NH_2_ protons, which were D_2_O exchangeable) 6.77–8.00 (m, 8H, Ar-H)

### 3.4. General procedure for the synthesis of 4-(2-methylquinoxalin-3-yloxy)-N-substituted benzylidine benzamines **7a–e**

A mixture of equalmolar quantities of compound **6** (0.01 M) and aromatic aldehydes (0.01 M) in ethanol (25 mL) containing a catalytic amount of glacial acetic acid were refluxed for sufficient time to complete the reaction, as monitored by TLC. The products so obtained were filtered, washed and recrystallized from appropriate solvents. 

#### 3.4.1. 4-[-{4-(2-Methylquinoxalin-3-yloxy)-phenyl}-iminomethyl]-phenol (**7a**)

IR (KBr, cm^−1^): 3,400 (phenol O-H), 3,065 (methyl C-H), 1,584, (-N=CH), 1,250 (ether C-O) and at 1,205 (phenol C-O). ^1^H NMR (CDCl_3_): 2.840 (s, 3H, CH_3_ attached to quinoxaline ring), 5.024 (s, 1H, Ar-OH), 6.861–8.052 (m, 12H, Ar-H), 9.031 (s, 1H, CH=N-). MS: m/z 355. Anal. Calculated for C_22_ H_17_ N_3_ O_2_: C, 74.35; H, 4.82; N, 11.82%.Found: C 74.15, H 5.00, N 12.05%.

#### 3.4.2. 4-(2-methylquinoxalin-3-yloxy)-N-(2-nitrobenzylidene)-benzamine (**7b**)

IR (KBr, cm^−1^): 3,060 (methyl C-H), 1,572 (-N=CH), 1,518 and 1,340 (aromatic nitro group -O-N=O & C-N), 1,223 (ether C-O). ^1^H NMR (CDCl_3_): 2.842 (s, 3H, CH_3_ attached to quinoxaline ring), 7.261–8.359 (m, 12H, Ar-H), 9.031, (s, 1H, CH=N-). MS: m/z 384. Anal. Calculated for C_22_ H_16_ N_4_ O_3_: C 68.74, H 4.20, N 14.58%.Found: C 68.58, H 4.00, N 14.75%. 

#### 3.4.3. 4-[-{4-(2-Methylquinoxalin-3-yloxy)-phenyleneimino}-methyl]-N-dimethylbenzamine (**7c**)

IR (KBr, cm^−1^): 2,928 (methyl C-H), 1,590 (-N=CH), 1,329 (tertiary amine C-N),1,226 (ether C-O). ^1^H NMR (CDCl_3_): 2.835 (s, 3H, CH_3_ attached to quinoxaline ring), 3.025 (s, 6H, CH_3_ protons of 3° amine), 7.028–8.212 (m, 12H, Ar-H), 8.516 (s, 1H, CH=N-). MS: m/z 382. Anal. Calculated for C_24_ H_22_ N_4_ O: C 75.37, H 5.80, N 14.65%.Found: C 75.60, H, 5.65, N 14.80%.

#### 3.4.4. [{4-(2-Methylquinoxaline-3-yloxy)-phenyleneimino}-methyl]-6-methoxyphenol (**7d**)

IR (KBr, cm^−1^): 3,450 and 1192 (phenol O-H & C-O), 3,060 (methyl C-H), 1,580 (-N=CH), 1,192 and 1,011 (methoxy C-O), 1223 (ether C-O). ^1^H NMR (CDCl_3_): 2.841 (s, 3H, CH_3_ attached to quinoxaline ring), 3.960 (s, 3H, –OCH_3_), 5.124 (s, 1H, phenolic–OH), 6.887–8.001 (m, 11H, Ar-H), 8.702 (s, 1H, CH=N-). MS: m/z 385. Anal. Calculated for C_23_ H_19_ N_3_ O_3_: C 71.67, H 4.97, N 10.90%. Found: C 71.56, H 4.80, N 11.05%.

#### 3.4.5. 4-(2-Methylquinoxalin-3-yloxy)-n-(3, 4, 5-trimethoxybenzylidene)-benzamine (**7e**)

IR (KBr, cm^−1^): 2,941, (methyl C-H), 1,579 (-N=CH), 1,199 and 999, (methoxy C-O), 1,226 (ether C-O). ^1^H NMR (CDCl_3_): 2.838 (s, 3H, CH_3_ attached to quinoxaline ring), 3.934–3.971 (t, 9H, OCH_3_), 6.887–7.998(m, 10H, Ar-H), 8.441 (s, 1H, CH=N-) group. MS: m/z 429. Anal. Calculated for C_25_ H_23_ N_3_ O_4_: C 69.92, H 5.40, N 9.78%.Found: C 70.20, H 5.15, N 9.95%. All the synthesized compounds were purified by recrystallization from appropriate solvent monitored by TLC. The list of all the synthesized compounds is shown in the Table 2.

### 3.5. Antibacterial Activity

The antibacterial activity was assayed by agar plate disc diffusion method [18] at the concentration of 50 µg per disk. All the synthesized compounds were tested *in vitro* for their antibacterial activity against microorganisms such as *Staphylococcus aureus*, *Bacillus subtilis* (gram positive), *Escherichia coli*, *and Pseudomonas aerugenosa* (gram negative) strains. Each test compounds were dissolved in dimethylsulphoxide (DMSO) to get a concentration of 10 mg/mL. The disc (6 mm in diameter) was impregnated with 5 µL of each test solution to get 50 µg/disc, air dried and placed on the agar medium, previously seeded with 0.2 mL of broth culture of each organism for 18 hours. The plates were incubated at 37 °C for 24 hours and the inhibition zones measured in mm. Discs impregnated with DMSO were used as a control and ciprofloxacin discs as antibacterial reference standard.

**Table 2:** Reaction conditions and physical data of synthesized compounds.

Compounds	R	Reaction Time	Crystallization Solvents	M.P. (°C)	Mobile phase	R_f_ value
**4**	-	30 hours	Ethanol	116–117	Ethyl acetate: *n*-Hexane (1:1)	0.72
**5a**	H	10 hours	Ethanol	140	Ethyl acetate: n-Hexane (1:1)	0.80
**5b**	2-Cl	17 hours	Ethanol	142	Ethyl acetate: n-Hexane (1:1)	0.78
**5c**	4-CH_3_	9 hours	Ethanol	159–160	Ethyl acetate	0.75
**5d**	4-COOH	16 hours	Ethanol	221–222	Ethyl acetate: n-Hexane (1:1)	0.50
**5e**	2-CH_3_,6-CH_3_	5 hours	Ethanol	131–132	Ethyl acetate	0.87
**6**	-	30 hours	Ethanol	178	Ethyl acetate: n-Hexane (1:1)	0.70
**7a**	4-OH	6 hours	Ethanol	220	Ethyl acetate: n-Hexane (1:1)	0.60
**7b**	2-NO_2_	1 hours	Ethanol	171–172	Ethyl acetate: n-Hexane (1:1)	0.77
**7c**	4-N(CH_3_)2	15 hours	Ethanol	220	Ethyl acetate: n-Hexane (1:1)	0.70
**7d**	2-OH,3-OCH_3_	1 hours	Ethanol	190	Ethyl acetate: n-Hexane (1:1)	0.85
**7e**	3,4,5(OCH_3_)_3_	21 hours	Ethanol	135	Ethyl acetate: n-Hexane (1:1)	0.65

### 3.6. Antifungal Activity

The antifungal activity [19] was assayed by the Sabouraud dextrose agar media plate disc diffusion method at a concentration of 50 µg per disk. All the synthesized compounds were tested *in vitro* for their antifungal activity against microorganisms such as *Aspergillus niger* and *Candida albicans*. Each test compound was dissolved in dimethylsulphoxide (DMSO) to get a concentration of 10 mg/mL. The disc (6 mm in diameter) was impregnated with 5 µL of each test solution to get 50 µg/disc; air dried and placed on the Sabouraud dextrose agar media, previously seeded with 0.2 mL of broth culture of each organism for 18 hours. The plates were incubated at 22 °C for 48 hours and the inhibition zones measured in mm. Discs impregnated with DMSO were used as a negative control and fluconazole discs as antifungal reference standard. The results have already been shown are in Table 1.
